# Targeting the Ubiquinol-Reduction (Q_i_) Site of the Mitochondrial Cytochrome *bc_1_* Complex for the Development of Next Generation Quinolone Antimalarials

**DOI:** 10.3390/biology11081109

**Published:** 2022-07-25

**Authors:** Kangsa Amporndanai, Nattapon Pinthong, Paul M. O’Neill, W. David Hong, Richard K. Amewu, Chandrakala Pidathala, Neil G. Berry, Suet C. Leung, Stephen A. Ward, Giancarlo A. Biagini, S. Samar Hasnain, Svetlana V. Antonyuk

**Affiliations:** 1 Molecular Biophysics Group, Institute of Systems, Molecular and Integrative Biology, Faculty of Health and Life Sciences, University of Liverpool, Liverpool L69 7ZB, UK; kangsa.amporndanai@Vanderbilt.Edu (K.A.); n.pinthong@liverpool.ac.uk (N.P.); s.s.hasnain@liverpool.ac.uk (S.S.H.); 2 Department of Biochemistry, Vanderbilt University School of Medicine, Nashville, TN 37232-0146, USA; 3 Department of Protozoology, Faculty of Tropical Medicine, Mahidol University, Bangkok 10400, Thailand; 4 Department of Chemistry, University of Liverpool, Liverpool L69 7ZD, UK; davidhwq@liverpool.ac.uk (W.D.H.); amewu@ug.edu.gh (R.K.A.); drchandrakalapidathala@cimedlife.com (C.P.); ngberry@liverpool.ac.uk (N.G.B.); scleung@liverpool.ac.uk (S.C.L.); 5 Department of Chemistry, School of Physical and Mathematical Sciences, University of Ghana, Accra P.O. Box LG 586, Ghana; 6 Composite Interceptive Med-Science Laboratories Pvt. Ltd., Bengaluru 60099, Karnataka, India; 7 Centre for Drugs and Diagnostics, Tropical Disease Biology, Liverpool School of Tropical Medicine, Liverpool L3 5QA, UK; steve.ward@lstmed.ac.uk (S.A.W.); giancarlo.biagini@lstmed.ac.uk (G.A.B.)

**Keywords:** antimalarial, *Plasmodium falciparum*, atovaquone, drug resistance, quinolone, *bc*_1_ inhibitor, crystallography, molecular modeling, homology modeling, mitochondria

## Abstract

**Simple Summary:**

Malaria is a life-threatening disease which infects millions of people a year via mosquito bites, particularly in developing countries. Although many malaria drugs are available in the market today, all of them have been challenged by drug-resistant variants. Developing a new drug to fight mutated malaria is extremely critical. Cytochrome *bc_1_* complex of malaria parasites is an important drug target focused on by several antimalarial development programs. One of those is the 4(1H)-quinolone series which inhibits cytochrome *bc_1_* and effectively kills drug-resistant malaria parasites. However, some of these compounds have unexpected toxicity due to cross-species inhibition of human cytochrome *bc_1_*. In this work, we explore by experimental and computational studies how 4(1H)-quinolone compounds work with human and parasite cytochrome *bc_1_*. This information reveals the key to improved selectivity between human and parasite cytochrome *bc_1_* and helps drug developers to design new compounds with better therapeutic efficiency and safety.

**Abstract:**

Antimalarials targeting the ubiquinol-oxidation (Q_o_) site of the *Plasmodium falciparum* bc_1_ complex, such as atovaquone, have become less effective due to the rapid emergence of resistance linked to point mutations in the Q_o_ site. Recent findings showed a series of 2-aryl quinolones mediate inhibitions of this complex by binding to the ubiquinone-reduction (Qi) site, which offers a potential advantage in circumventing drug resistance. Since it is essential to understand how 2-aryl quinolone lead compounds bind within the Qi site, here we describe the co-crystallization and structure elucidation of the bovine cytochrome *bc_1_* complex with three different antimalarial 4(1H)-quinolone sub-types, including two 2-aryl quinolone derivatives and a 3-aryl quinolone analogue for comparison. Currently, no structural information is available for *Plasmodial* cytochrome *bc_1_*. Our crystallographic studies have enabled comparison of an in-silico homology docking model of *P. falciparum* with the mammalian’s equivalent, enabling an examination of how binding compares for the 2- versus 3-aryl analogues. Based on crystallographic and computational modeling, key differences in human and *P. falciparum* Q_i_ sites have been mapped that provide new insights that can be exploited for the development of next-generation antimalarials with greater selective inhibitory activity against the parasite *bc_1_* with improved antimalarial properties.

## 1. Introduction

Malaria remains a devastating disease: it was estimated that in 2020 there were ~241 million cases and 627,000 deaths, primarily in children under 5 years old. Although much progress has been made to reduce the rate of malaria mortality and morbidity [1], multi-drug resistance is now widespread with the gradual spread of Artemisinin-based combination therapy (ACT) resistance being a major concern [2]. This has mobilized a coordinated global effort to replenish the drug development pipeline with new drug candidates that are able to overcome existing parasite drug resistance [3].

The mitochondrial electron transport chain of the malaria parasite is a validated target for antimalarial drug development [4]. Cytochrome *bc_1_* is a multi-subunit protein complex embedded in the inner mitochondrial membrane [5] as part of the mitochondrial electron transport chain. It has three catalytic subunits: cytochrome *b*, cytochrome *c_1_*, and the Rieske iron-sulfur protein, which are responsible for proton translocation through the membrane and electron transfer to cytochrome *c* via the redox reaction of ubiquinone in the Q cycle [6]. The Q cycle takes place in two distinct binding sites on the cytochrome *b* subunit; the ubiquinol-oxidation (Q_o_) site and the ubiquinone-reduction (Q_i_) site [7]. In parasites, cytochrome *bc_1_* has a critical function to re-generate ubiquinone for dihydroorotate dehydrogenase (DHODH), which is an important enzyme in parasites’ de novo pyrimidine biosynthesis pathway [8,9,10]. Their role in electron transport chains makes them an attractive target for drug development for various apicomplexan parasites, including *Toxoplasma gondii*, *Trypanosoma cruzi*, and *Leishmania donovani* [11,12]. Atovaquone (used in combination with Proguanil, e.g., Malarone ^TM^) is a hydroxynaphthoquinone and is widely-used in the treatment and prevention of uncomplicated malaria [13,14,15]. Atovaquone can eliminate malaria (*Plasmodium spp.*) and toxoplasmosis (*Toxoplasma gondii*) [16] parasites in multiple stages of the life cycle by inhibiting the Q_o_ site of cytochrome *bc_1_* and ultimately disrupting de novo pyrimidine biosynthesis [17]. Point-mutations within the Q_o_ site, especially Y268S, weaken the binding of atovaquone to the Q_o_ pocket, leading to the emergence of atovaquone-resistant malaria strains [18,19].

Structures of cytochrome *bc_1_* from various species have been determined by X-ray crystallography [20,21,22,23,24,25,26] and cryo-electron microscopy [27,28,29]. Inhibitor-bound protein structures show that most inhibitors bind to the Q_o_ site [25,30,31]. In 2015, the Q_i_ site from bovine cytochrome *bc_1_*, a human surrogate with 80% identity with cytochrome *b* [32], was crystallographically shown as an alternative binding site for antimalarial 4(1H)-pyridones, GSK932121, and GW844520 [33]. Both 4(1H)-pyridone compounds have outstanding antimalarial properties and were positioned for preclinical candidate selection; however, both compounds were withdrawn due to toxicity issues discovered in preclinical studies [34,35]. The crystallographic studies suggested that this class of molecules has the propensity to bind to the mammalian *bc_1_* complex at the Q_i_ site and may be the cause of toxicity. However, a direct link of such a binding to the toxicities observed in preclinical studies needs further investigation. Nevertheless, selectivity between the host and plasmodial targets is a key element in safe-drug design, so understanding differences between the mammalian and parasite Q_i_ targets is a key objective for rational drug design.

**Figure 1:**
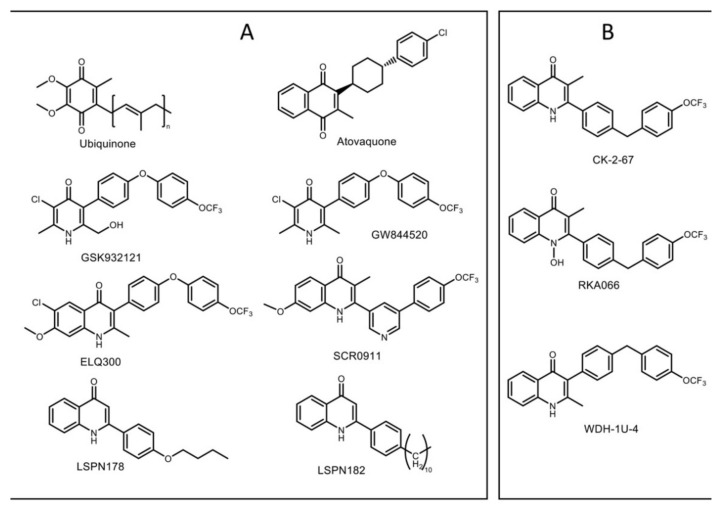
Chemical structures of cytochrome *bc_1_* ligands. (**A**) Ubiquinone, a native substrate that binds to both Q_o_ and Q_i_ sites of cytochrome *bc_1_*; atovaquone, a hydroxy-naphthoquinone targeting the Q_o_ site; GSK932121 and GW844520, 4(1H)-pyridone-based compounds developed by GlaxoSmithKline; quinolone-based compounds ELQ300 and SCR0911, lead antimalarial compounds; LSPN 178 and LSPN 182, modified 2-quinolone that inhibits both stages of the parasite; (**B**) CK-2–67, RKA066, and WDH-1U-4 were the 4(1H)-quinolone-based compounds that were co-crystallized with bovine cytochrome *bc_1_* in this study.

Chemical compounds based on the 4(1H)-quinolone template have been developed as antimalarials for three quarters of the century [36]. The design of these compounds has been based on the use of modified ubiquinone-like scaffolds that target cytochrome *bc_1_* [36]. The discovery and development of endochin-like-quinolones (ELQs) have attracted much interest in the field of malaria drug discovery [37] (Figure 1a). A front runner, ELQ300, has been developed as an inhibitor of cytochrome *bc_1_*. ELQ300 is reported to be highly selective for *Plasmodium bc_1_* over human *bc_1_* and displays potent antimalarial activity against blood stages (and a number of other life-cycle stages) of *P. falciparum*, including atovaquone-resistant strains [38,39]. A pro-drug of ELQ300 is currently progressing in preclinical development [40]. Recent modification of 4(1H)-quinolones, LSPN 182, and LSPN 178, where position 2 of the quinolone ring was substituted, showed a high potency in the inhibition of the sexual and asexual stage of the parasite [41] (Figure 1A). This finding highlights the significant potential of 4(1H)-quinolones as a scaffold for the development of antimalarial drugs.

Recently, 2-pyridyl substituted 4(1H)-quinolone SCR0911 (Figure 1A) was reported, featuring the replacement of 3-aryl sidechain with 2-pyridyl sidechain [32]. This modification provided higher aqueous solubility (7.8 µM at pH 1) than ELQ300 (<1 µM at pH 1) and high potency against drug-resistant *P. falciparum* in the low nanomolar IC_50_ range (concentration of drug required to achieve half-maximal growth suppression) (Table 1). The specificity to Q_o_/Q_i_ site of 4(1H)-quinolone-based compounds was determined by monitoring cytochrome *bc_1_* activity in Q_o_/Q_i_ site in yeast mutants [42,43] and *P. falciparum* [44,45]. Subtle substituent changes in 4(1H)-quinolone-based compounds can affect Q_o_ or Q_i_ binding preference. The binding of SCR0911 to bovine cytochrome *bc_1_* was visualized by X-ray crystallography and cryo-electron microscopy [29], revealing that SCR0911 behaved as a selective Q_i_ binder in crystal and in-solution studies. In parallel, in vitro assessments of 4(1H)-quinolone derivatives, such as CK-2-67, RKA066, and WDH-1U-4 (Figure 1B), were reported to possess inhibitory activities against both cytochrome *bc_1_* and the *P. falciparum* mitochondrial type II NADH: ubiquinone oxidoreductase (*Pf*NDH_2_) [10,46] (Table 1). *Pf*NDH_2_ is a single-subunit enzyme in the mitochondrial electron transport chain of *P. falciparum* that acts as an alternative enzyme for typical complex I [47]. It enables uncoupled oxidation of NADH that contributes to the regeneration of ubiquinol for cytochrome *bc_1_* [48]. Selectivity of inhibitors for the two enzymes is affected by manipulating the 2- or 3-aryl substituent. The 3-aryl-4(1H)-quinolones, such as WDH-1U-4 and ELQ300, display stronger levels of inhibition against *Pf* cytochrome *bc_1_* [10,38], while the 2-aryl-4(1H)-quinolones, such as CK-2-67 and RKA066, can also target *Pf*NDH_2_ [10,46].

We co-crystallized bovine cytochrome *bc_1_* with three antimalarial 4(1H)-quinolones, CK-2-67, RKA066, and WDH-1U-4, to investigate the binding modes of these inhibitors within the Qi site of representatives of 2-aryl and 3-aryl quinolone. Due to the lack of *bc_1_* structure from *P. falciparum*, we generated the homology model of *Pf* cytochrome *bc_1_* and explored possible inhibitor binding modes of these antimalarial compounds by using molecular docking.

## 2. Materials and Methods

### 2.1. Cytochrome bc_1_ Purification

Cytochrome *bc_1_* was purified from mitochondria, as previously described [29]. Crude mitochondria were isolated from fresh bovine hearts [49] and solubilized with the addition of dodecyl-maltoside (DDM). The solubilized protein was loaded onto a 50 mL DEAE Sepharose CL6B (GE Healthcare, Uppsala, Sweden) column pre-equilibrated in and washed with three column volumes of 50 mM KPi pH 7.5, 250 mM NaCl, 0.5 mM EDTA, and 0.01% DDM. The bound protein was eluted along a linear gradient from 250 to 500 mM NaCl. Cytochrome *bc_1_* fractions were pooled and concentrated in a centrifugal ultrafilter (MWCO. 100 kDa) and loaded onto a 120 mL Sephacryl S300 column (GE Healthcare, Uppsala, Sweden) equilibrated in and eluted at a flow rate of 0.5 mL/min with 20 mM KMOPS pH 7.2, 100 mM NaCl, 0.5 mM EDTA, 0.01% DDM. Cytochrome *bc_1_* fractions were pooled and concentrated to 40 mg/mL. PEG fractionation with an increasing amount of PEG4000 was used to precipitate cytochrome *bc_1_*. The protein started precipitating at 2% PEG4000, and pure protein was in the fraction of 2.5–4% PEG4000. Cytochrome *bc_1_* pellet was re-solubilized in 25 mM KPi pH 7.5, 100 mM NaCl, 0.5 mM EDTA, and 0.015% DDM and dialyzed in the same buffer in a centrifugal ultrafilter to remove residual PEG. The inhibitors were dissolved in DMSO at the concentration of 50 mM and 5 μM cytochrome *bc_1_* was incubated with 50 μM inhibitor (10-fold molar excess) at 4 °C for 12 h.

### 2.2. Crystallization, Data Collection, and Structure Refinement

The inhibitor-bound cytochrome *bc_1_* was mixed with 1.6% HECAMEG and adjusted to the protein concentration of 40 mg/mL. In total 2 μM of final protein solution was mixed with 2 μM of reservoir solution (50 mM KPi pH 6.8, 100 mM NaCl, 3 mM NaN_3_, 10–12% PEG4000). The crystals were grown using the hanging drop method at 4 °C to the mature size of ~100 μm within four days. The single crystals were soaked stepwise in 50% ethylene glycol with reservoir solution prior to freezing in liquid nitrogen. Two datasets *bc_1_*-CK-2-67 and *bc_1_*-RKA066 were collected at PROXIMA-1 beamline (SOLEIL, Paris, France) at 100 K, using 0.9786 Å wavelength on EIGER-X 16 M detector and 0.9786 Å wavelength on Pilatus3 6M, respectively, *bc_1_*-WDH-1U-4 data were collected at PROXIMA-2A beamline with 0.9800 Å wavelength using EIGER X 9M detector (Paris, France). All data were processed by iMosflm [50] and scaled by Aimless [51]. The structures were refined by jelly body and TLS refinement in Refmac5 [52] using bovine cytochrome *bc_1_* (PDB:5OKD) without inhibitors or ligands as the starting model. The model was manually rebuilt in COOT [53] between the refinement cycles. The inhibitor molecules were produced by using Jligand [54]. Data collection and refinement statistics are summarized in Table 1.

### 2.3. Parasite Culture and Drug Sensitivity Measurements

*P. falciparum* 3D7 parasites were cultured in human erythrocytes (O, Rh^+^) at 5% haematocrit under 5% CO_2_, 2% O_2_, and 93% N_2_. Cultures were grown in complete medium (CM) containing RPMI 1640 medium (Gibco) supplemented with 25 mM HEPES (VWR), 50 μg/mL hypoxanthine, 0.25 mM NaHCO_3_, 50 μg/mL gentamicin sulfate, and 10% pooled heat inactivated AB + human serum [55]. Parasite growth was synchronized by treatment with sorbitol [56]. Drug susceptibilities were assessed by the measurement of fluorescence after the addition of SYBR Green I, as previously described [57]. Drug IC_50_ values were calculated from the log of the dose/response relationship, as fitted with Grafit software version 7.0.3 (Erithacus Software, Kent, UK).

### 2.4. Characterisation of Compounds and Their Purity

CK-2-67, RKA066 and WDH-1U-4 were synthesized as described by Pidathala C. et al. [46].

The structures of these three compounds were confirmed by ^1^H NMR, as follows, which matched previous report values.

CK-2-67: ^1^H NMR (400 MHz, DMSO) δH 8.98 (s, 1H, NH), 8.27 (d, *J* = 8.3 Hz, 1H), 7.60 (d, *J* = 8.1 Hz, 1H), 7.56 (dt, *J* = 1.4 Hz, 8.3 Hz, 1H), 7.9 (d, *J* = 8.1 Hz, 2H), 7.26 (dt, *J* = 1.5 Hz, 8.1 Hz, 1H), 7.20 (d, *J* = 8.0 Hz, 2H), 7.16 (d, *J* = 8.6 Hz, 2H), 7.11 (d, *J* = 8.1 Hz, 2H), 3.96 (s, 2H), 2.01 (s, 3H);

RKA066: ^1^H NMR (400 MHz, DMSO) δ 11.20 (s, 1H), 8.23 (d, *J* = 8.0 Hz, 1H), 7.84 (d, *J* = 7.9 Hz, 1H), 7.73 (t, *J* = 8.1 Hz, 1H), 7.46 (d, *J* = 8.3 Hz, 2H), 7.44 (d, *J* = 8.0Hz, 2H), 7.39 (t, *J* = 7.9 Hz, 1H), 7.35 (d, *J* = 8.1 Hz, 2H), 7.32 (d, *J* = 7.9 Hz, 2H), 4.07 (s, 2H),1.75 (s, 3H).

WDH-1U-4: ^1^H NMR (400 MHz, DMSO) δ 11.60 (s, 1H), 8.05 (d, *J* = 6.8 Hz, 1H), 7.61 (t, *J* = 6.9 Hz, 1H), 7.51 (d, *J* = 8.0 Hz, 1H), 7.44–7.38 (m, 3H), 7.33–7.21 (m, 4H), 7.16 (d, *J* = 8.1 Hz, 2H), 4.00 (s, 3H), 2.20 (s, 3H).

All three compounds showed >95% purity when tested by the HPLC method described here. HPLC analysis was carried out using the Agilent 1200 system. The HPLC method used the following conditions: ZORBAX Eclipse Plus C18 (4.6 mm × 100 mm, 3.5 µm) at 25 °C with 1.0 mL/min flow rate; solvents: (A) water containing 0.05% trifluoroacetic acid and (B) acetonitrile containing 0.05% trifluoroacetic acid; method (Acidic, 2–98%): run time: 15 min, gradient: 2% B hold to 2 min, 2–98% B in 10 min, then hold at 98% B to 15 min.

### 2.5. Plasmodium Falciparum Homology Model Generation

The primary sequence of *P. falciparum* is retrieved from UniProtKB (ID: Q02768). *Pf* cytochrome *b* homology model was generated using web-based protein modelling server: SwissModel software (Swiss Institute of Bioinformatics and Biozentrum, Basel, Switzerland) [58,59,60] with the bovine cytochrome *b* template (chain c of 5OKD model). The model was built based on target-template alignment using ProMod3 version 1.1.0 (Swiss Institute of Bioinformatics and Biozentrum, Basel, Switzerland) [61]. The conserved residues were preserved in the model, while sidechains differences due to differences in the primary sequences were re-modelled using a fragment library. The final model was regularized by a force field and its quality assessed using the QMEAN scoring [62]. The *Pf* homology model used in this paper has a QMEAN score of −5.67 and 41.71% sequence identity.

### 2.6. Molecular Docking

Full details of molecular docking to the P. falciparum homology model are provided in supplementary information. In brief, the quinolone lead compounds docking was carried out by web-based docking platform: SwissDock software SwissDock (Swiss Institute of Bioinformatics and Biozentrum, Basel, Switzerland) [63] based on EADock DSS using the CHARMM force field [64]. The docking was performed within the defined *Pf* Q_i_ site, using the “Accurate” parameter, allowing flexibility for the sidechain within 5 Å to its reference binding mode. The final solutions were determined based on binding pose corresponding to the solved bovine crystal structure with the highest *Fullfitness* score (Table S1). All non-covalent interactions between the inhibitor molecule and Q_i_ site residues were visualized by ViewContacts version 2018 software (DesertScientific, Sydney, Australia) [65].

### 2.7. Bovine Cytochrome bc_1_ Activity Assay

Bovine cytochrome *bc_1_* inhibition assays were measured in triplicate in 50 mM KPi pH 7.5, 2 mM EDTA, 10 mM KCN, 30 μM equine cytochrome *c* (Sigma-Aldrich, St. Louis, MO, USA), and 2.5 nM bovine cytochrome *bc_1_* at room temperature. The inhibitors were added to the assay without prior incubation. The reaction was initiated by the addition of 50 μM decylubiquinol (Sigma-Aldrich, St. Louis, MO, USA). The reduction of cytochrome *c* was monitored by the different absorption between 550 and 542 nm using extinction coefficient of 18.1 μM^−1^cm^−1^ in a SPECTRAmax Plus 384 UV-visible Spectrometer (Molecular Devices, San Jose, CA, USA).

## 3. Results

### 3.1. Inhibitory Effect of 4(1H)-Quinolones Binding to the Qi Site of Bovine Cytochrome bc_1_

Antimalarial activity profiles of studied 4(1H)-quinolones and 4(1H)-pyridones against drug-sensitive (3D7) and atovaquone-resistant *P. falciparum* (TM90C2B) were reported in previous studies and are summarized in Table 1 [10,32,34,38,46]. A low inhibition of bovine *bc*_1_ represents a potential for increased mammalian/human safety. The bovine *bc_1_* inhibition level of studied 4(1H)-quinolones and 4(1H)-pyridones were measured in this work. Amongst all the compounds tested, CK-2-67 and RKA066 has the strongest bovine *bc_1_* inhibition at 0.1 µM (90% and 81%, respectively) compared to WDH-1U-4 (7%). Meanwhile, the pyridone GSK932121 showed a 65% inhibition in bovine *bc*_1_ at 0.1 µM, suggesting that the presence of the NH and N-OH group within the core may enhance mammalian *bc_1_* inhibition, leading to poor selectivity (Table 1).

### 3.2. Binding of 4(1H)-Quinolones to the Qi Site of Bovine Cytochrome bc_1_

**Figure 2:**
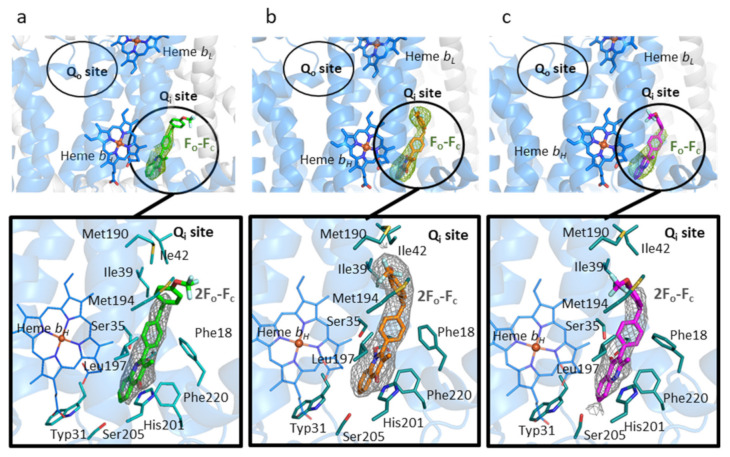
The cytochrome *bc_1_* Q_i_ site with bound inhibitors (**a**) CK-2-67, (**b**) RKA066, and (**c**) WDH-1U-4 molecules are shown as green, orange, and magenta sticks, respectively. Upper insert: the omit F_o_-F_c_ map is contoured at 3σ level and shown as green mesh. The Q_i_ and Q_o_ sites are marked by black circles. Lower insert: the molecules are shown as sticks surrounded by 2F_o_-F_c_ electron density contoured at 1σ level. Q_i_ site residues are shown as blue sticks, hydrogen bonds as black dash lines. The cartoon representation of bovine cytochrome *b* subunit is shown in blue.

X-ray crystallographic structures were obtained of bovine cytochrome *bc1* co-crystallized with CK-2-67, RKA066, and WDH-1U-4 in the resolution range of 3.3–3.5 Å (Table 2). Omit Fo-Fc electron density map revealed clear electron density in the Q_i_ site region near heme b_H_, enabling the placement of each inhibitor molecule in the corresponding structures. No additional electron density was found within the Q_o_ site near heme b_L_, establishing binding of these inhibitors unambiguously to the Q_i_ site only, though the trifluoromethoxy phenyl sidechain of CK-2-67 and WDH-1U-4 was partially visible in the refined 2F_o_-F_c_ electron density (Figure 2).

**Figure 3:**
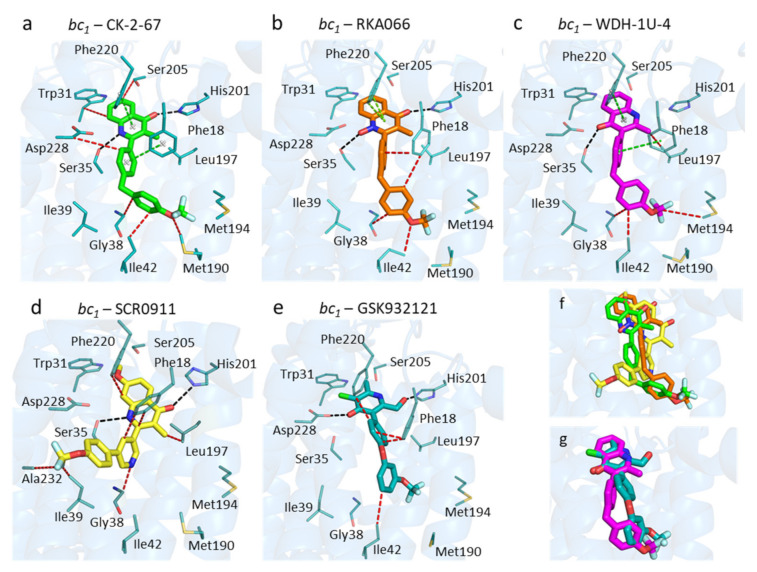
Binding modes of selected antimalarial compounds within the bovine Q_i_ site. (**a**) CK-2-67 (green sticks). (**b**) RKA066 (orange sticks). (**c**) WDH-1U-4 (magenta sticks). (**d**) SCR0911 (yellow sticks) from the 5OKD structure. (**e**) GSK932121 (teal sticks) from the 4D6U structure. (**f**) Superimposed crystal structures with 2-aryl 4(1H)-quinolones: SCR0911, CK-2-67, and RKA066. (**g**) Superimposed crystal structures of WDH-1U-4 and GSK932121. Black, cyan, green, and red dashes represent hydrogen bonds, electrostatic, aromatic-aromatic, and hydrophobic interactions between ligands and protein residues, respectively. The blue cartoon represents the bovine cytochrome *b* subunit.

The amino acid residues in the Q_i_ site adopt the same arrangement regardless of the inhibitor within (Figure 3). Unexpectedly, binding modes of CK-2-67, RKA066, and WDH-1U-4 within the Q_i_ pocket appear to be similar, even though they are substituted at different positions on the quinolone headgroup, i.e., 2- and 3-positions (Figure 3a–c). For all three analogues, the 4(1H)-quinolone head group is placed in the hydrophilic region between His201 and Asp228 and forms edge-to-face aromatic interaction with Phe220.

The bis-aryl sidechain in the 4(1H)-quinolone analogues extends away from head group into hydrophobic cavity between Phe18, Gly38, Ile39, and Ile42 residues, and the trifluoromethoxy group is positioned between Met190 and Met194.

The 4(1H)-quinolone ring orientation in the 3-aryl-4(1H)-quinolone (WDH-1U-4) is similar to the orientation of the 4(1H)-pyridone ring in GSK932121 but lies in a reversed direction to the other 2-aryl-4(1H)-quinolones studied (Figure 3c,e,g) (i.e., the 4(1H)-quinolone head of WDH-1U-4 is flipped-over by 180° from the 4(1H)-quinolone head of CK-2-67 and RKA066, enabling a single hydrogen bond between carbonyl and Ser35). The 4(1H)-quinolone ring ligand bound complex also maintains an H-bonding distance for His201.

In addition to the inversion of the quinolone head group for the 3-aryl analogue WDH-1U-4, there are additional differences in the finer details when compared to previously reported 2-aryl quinolone binders of the Q_i_ site, (SCR0911 and GSK932121). The more flexible sidechain, 2-aryl-4(1H)-quinolones, of CK-2-67 and RKA066 has the carbonyl group closer to His201, and the NH group is closer to Asp228 (Figure 3a,b,d,f). The CK-2-67 and RKA066 molecules form two hydrogen bonds with Ser35 and His201 (Figure 3a,b). CK-2-67 forms hydrogen bonds between its carbonyl group to Nε2His201 and NH group to OγSer35 (Figure 3a). The functional group of CK-2-67 maintains a similar position of 4(1H)-quinolone to that of RKA066 pose (Figure 3f). RKA-066 also makes two hydrogen bonds; one is from the carbonyl group to Nε2His201 and the other is between the N-hydroxyl group and OγSer35. It also creates aromatic interaction with Phe18 and Phe220 in a face-to-edge manner (Figure 3a). In comparison to RKA066, CK-2-67 had stronger inhibitory effect against bovine *bc_1_* at 0.1 µM (90%, Table 1).

### 3.3. Predicted Binding Mode of 4(1H)-Quinolones within the Plasmodium Falciparum Q_i_ Site

**Figure 4:**
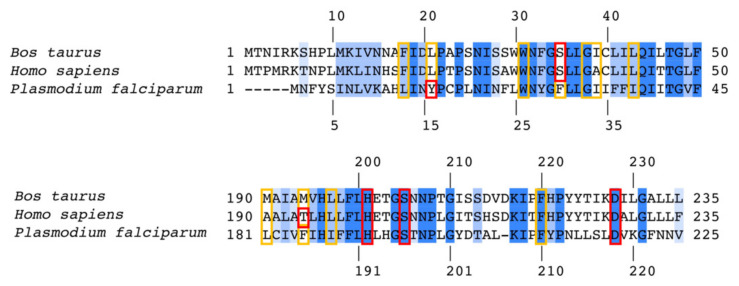
Sequence alignment between bovine, human, and *P. falciparum* cytochrome *b* showing conservation in the Q_i_ site. Fully and partially conserved residues are colored in deep and light blue, respectively. The numbering shown at the top and bottom corresponds to bovine (human) and *P. falciparum* sequences, respectively. The polar and non-polar residues interacting with 4(1H)-quinolones in crystal structures and docking models are highlighted in red and yellow boxes, respectively. Sequence identity between human and bovine cytochrome *b* is 93%.

The active sites of *Pf bc_1_* have significantly different sequences compared with human sequences (65% and 39% identity for Q_o_ and Q_i_ site, respectively). The sequence of *Pf* cytochrome *b* has a number of unique features from human sequences that may enable drug selectivity (Figure S1). A molecular docking study was carried out by an automated molecular-docking web-based tool: SwissDock [29,63] with focus on the Q_i_ site generated homology model of *Pf bc_1_*. The best conformation of each compound was determined based on the poses in the bovine crystal structure with the highest *FullFitness* scores (Table S1). The *Pf* Q_i_ binding pocket is smaller than the bovine Q_i_ site due to a shorter protein sequence (Figure 4). The inhibitors would have to adopt other poses in the *Pf bc_1_* Q_i_ site to avoid unfavorable steric clashes.

**Figure 5:**
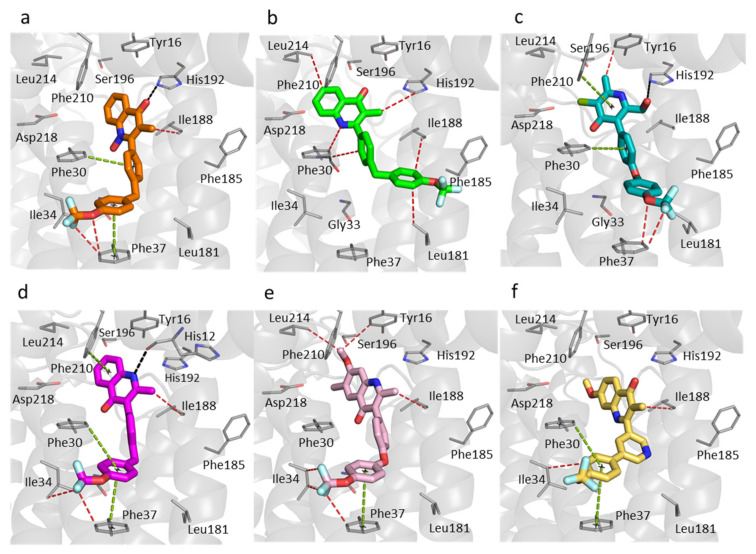
Molecular docking within the Q_i_ site of *P. falciparum* cytochrome *bc_1_*. The predicted binding mode of each compound. (**a**) RKA066 (orange sticks). (**b**) CK-2-67 (green sticks). (**c**) GSK932121 (teal sticks). (**d**) WDH-1U-4 (magenta sticks). (**e**) ELQ300 (pink sticks). (**f**) SCR0911 (yellow sticks). Black, cyan, green, and red dashes represent hydrogen bonds, electrostatic, π-π stacking, and hydrophobic interactions between ligands and protein residues, respectively. The grey cartoon represents bovine cytochrome *b* subunit.

The molecular docking indicated that all studied compounds could fit their 4(1H)-quinolone core into the apex of Q_i_ pocket between His192 and Asp218 and pack the trifluoromethoxy phenyl ring into the cavity between Phe30 and Phe37 via strong π-π stacking interactions (Figure 5). The *N*-hydroxyl group of RKA066 encourages the carbonyl group to form a hydrogen bond with His192 (Figure 5a).

Only two molecules, CK-2-67 and GSK932121, are not able to create strong π-π stacking contacts between the trifluoromethoxy phenyl rings and Phe30 and Phe37 (Figure 5b,c). CK-2-67 was less effective in parasite inhibition than the other compounds that were tested. It was the least active compound against *Pf bc_1_*, which is consistent with the molecular docking results, which showed only hydrophobic interactions and the lack of hydrogen bonds or aromatic stacking. In contrast, CK-2-67 and GSK932121 can form crucial hydrogen bonds between the hydroxyl methyl group and His192 displayed higher potency in both phenotypic and enzymatic *Pf bc_1_* assays than CK-2-67 (Table 1).

The 4(1H)-quinolone core of WDH-1U-4 was predicted to be at a hydrogen bonding distance to the main chain oxygen of His12 with the trifluoromethoxy phenyl sidechain of this compound forming favorable interactions with Tyr16, Phe30, Ile34, Phe37, and Phe210 (Figure 5d). This ligand pose of WDH-1U-4 explains potentially strong binding to the *Pf* Q_i_ site, which agrees with the potency of this compound (Table 1). Notably, ELQ300 was also reported to be highly active against *Pf bc_1_*, with an IC_50_ less than 1 nM (Table 1), and it displays similar interactions with Ile34 and Phe37 as WDH-1U-4. The docking results of ELQ300 and SCR0911 showed no hydrogen bond formation between protein and ligand within the *Pf* Q_i_ site (Figure 5e,f). The 7-Methoxy group in ELQ300 has a strong electrostatic interaction with Ser196 (Figure 5e), and the phenyl-pyridyl sidechain of SCR0911 possesses tight π-π stacking with aromatic sidechain of Phe30 and Phe37 (Figure 5f). These interactions could encourage binding of both compounds to the *Pf* Q_i_ pocket even without a predicted hydrogen bond, as the 4(1H)-quinolone cores of ELQ300 and SCR0911 are located close to His192 in this in silico analysis.

The 4(1H)-quinolones with bis-aryl tail (CK-2-67, RKA066, and WDH-1U-4, which have a methylene linker between phenyl rings) displayed weaker inhibition of *Pf* parasite growth compared to the 4(1H)-quinolones with phenoxy-phenyl (ELQ300) and phenyl-pyridyl sidechain (SCR0911) (Table 1). This trend was confirmed in *Pf* parasites with Q_o_ site mutations (TM90C2B), where CK-2-67 and RKA066 have low potency with triple-digit nanomolar IC_50_, while ELQ300, SCR0911, and WDH-1U-4 retained their potency.

To further confirm the relationship of chemical structure with Q_o_/Q_i_ site selectivity, the availability of either an inhibitor-bound *Pf bc_1_* structure or *P. falciparum* assay of Q_o_/Q_i_ mutated strains could be beneficial. However, in the absence of those, this computational study brought us to the conclusion that the flexibility of aromatic side chain could be a factor affecting the binding affinity to *Pf* Q_i_ site. It is clear that the rigid phenyl-pyridyl sidechain of SCR0911 forms strong π-π stacking with both Phe30 and Phe37 (Figure 5f) and as such it could be a good template for future compounds with enhanced selectivity for the Q_i_ site of *Pf bc_1_*.

### 3.4. Suggestions for Future Development of 4(1H)-Quinolones

**Figure 6:**
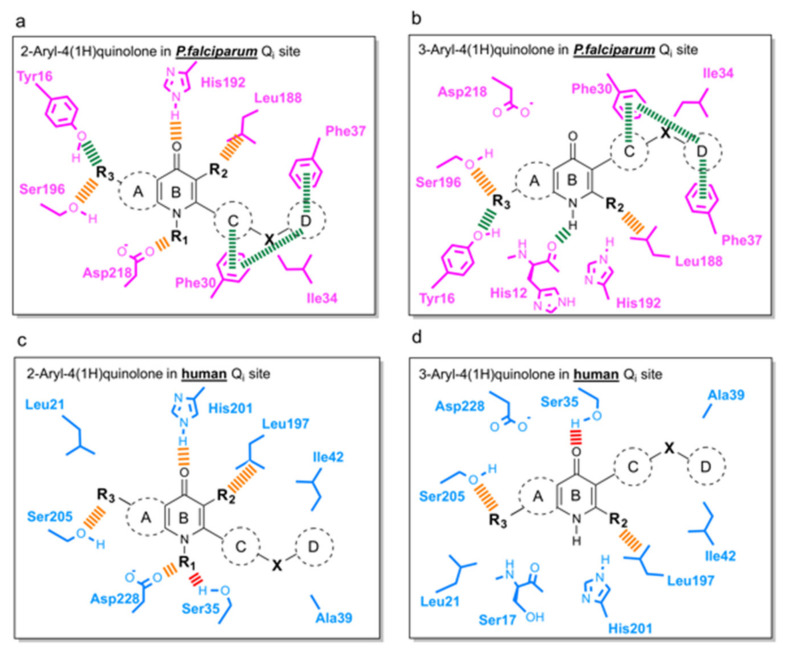
Schemes for the future development of aryl-4(1H)-quinolones targeting the Q_i_ site binding. (**a**) 2-Aryl-4(1H)-quinolones in *P. falciparum bc_1_*. (**b**) 3-Aryl-4(1H)-quinolone in *P. falciparum bc_1_*. (**c**) 2-Aryl-4(1H)-quinolones in human *bc_1_*. (**d**) 3-Aryl-4(1H)-quinolone in human *bc_1_* (based on PDB: 5XTE). Colored dashed bars represent the interactions that could be considered for lead compound design: green—the interactions observed only in *P. falciparum*; orange—the interactions observed in both species; red—the interactions observed only in humans.

Following the determination of co-crystallographic bovine *bc_1_*-compound structures and development of homology models of the *Pfbc_1_* active site, the final stage of our analysis involved mapping important protein-ligand interactions within *Pf* and the human Q_i_ site (Figure 6). Interactions with Phe30, Phe37, and Tyr16 residues (conserved only in *P. falciparum* (Figure 6a,b) are essential for binding selectively to *Pf bc_1_*. The aromatic ring D should be maintained for π-π stacking with Phe30 and Phe37, and hydrogen bond acceptors on ring A (R_3_), such as the methoxy group, could be added to assist hydrogen bond formation with Tyr16. As incorporation of 3-ester in a similar series of 4(1H)-quinolones always delivered potent antimalarial activity [66], the methyl group on ring B (R_2_) in our series could be replaced by a larger alkyl group, such as ethyl or *iso*-propyl, which could fill the space near the Leu188-like 3-ester group with more hydrophobic interactions. In order to minimize toxicity in the host (Figure 6c,d), growing functional groups at the NH (R_1_) should minimize electrostatic interaction between 4(1H)-quinolone head and Ser35 of human *bc_1_*. RKA066 (R_1_: OH) is an example, where R_1_ tightly binds to Asp218 of *Pfbc_1_* but has cross-reactivity to human cytochrome bc_1_ due to hydrogen bonds with Ser35. However, *N*-methyl quinolone was reported to be less effective than its counterpart to inhibit *P. falciparum* [67]. Careful considerations are needed when addressing R_1_ to develop new compounds. Strong intermolecular interactions between 4(1H)-quinolone rings can cause poor aqueous solubility, which hampers the development of new compounds. To increase solubility and pharmacological success of the new compounds, ring A or ring C could be replaced by saturated heterocycles, including a degree of polarity and reducing flatness of the molecule [68].

## 4. Discussion

Cytochrome *bc_1_* from bovine and humans have high sequence conservation for both Q_o_ and Q_i_ sites: 94% and 80%, respectively. The superimposed crystal structures of bovine [69] and human cytochromes *bc_1_* [28] showed that the residues in the Q_o_ and Q_i_ sites that interact with inhibitors are well-conserved between both species (Figure S2). Due to high homology between bovine and human proteins, bovine cytochrome *bc_1_* was used as a human surrogate in enzymatic and structural studies to determine possible toxicity through cross-reactivity of antimalarials against the human enzyme [29,32,33,70]. Crystal structures of ligand-bound bovine cytochrome *bc_1_* provide a powerful tool for visualizing in-situ ligand engagement with the target that is helpful for understanding how antimalarials inhibit mammalian *bc_1_* and optimize the compounds with improved safety profiles [33,70].

The electron density of the 4(1H)-quinolones inhibitors validated their position within the bovine Q_i_ site. The second aromatic ring of the bis-aryl tail, on the other hand, is less visible due to the flexibility of the sidechain, as seen previously for a 3-aryl sidechain quinolone compound, JAG21 [71]. This flexibility of the sidechain (phenoxyaryl) was also demonstrated in a previous investigation of the electron microscopy-derived structure of bovine *bc*_1_-GSK932121, where two distinct conformations of GSK932121 were seen in solution [29]. This flexibility of the trifluoromethoxy-substituted phenyl ring around the linker atom causes the inhibitors to adopt different conformations within the Q_i_ site. Consequently, it was difficult to position the second phenyl ring of CK-2-67 and WDH-1U-4 in the poorly defined electron density.

His201 and Asp228 are key amino acid residues in the Q_i_ pocket, which ubiquinone binds before reducing to ubiquinol within the Q cycle [22]. Some other known Q_i_ inhibitors, i.e., antimycin [30,69], NQNO [30], and ascochlorin [72], form hydrogen bonds to Asp228, His201, and Ser205 that block the Q_i_ pocket and ultimately disrupt the Q cycle. The additional hydrogen bonds between the quinolone core to His201 and Ser35 make CK-2-67 and RKA066 a more potent inhibitor of bovine *bc_1_*. Moreover, the presence of NH and N-OH in the core of CK-2-67 and RKA066 might enhance the inhibition of mammalian *bc_1_*, leading to poor selectivity. For the pyridone GSK932121, there is an additional hydrogen bond between its hydroxyl-methyl group and His201. This additional H-bonding likely plays a role in the strong inhibitory effects seen versus the bovine *bc_1_* complex of 65% at 0.1 µM. The results confirm that including the *N*-hydroxy and methoxy sidechains increases drug solubility. The poor physicochemical properties associated with these molecules may lead to greater off-target toxicity, thereby reducing the potential therapeutic index.

The difference in ligand poses and the number of hydrogen bonds between molecule and protein determines their affinity for bovine cytochrome *bc_1_* and explains how some compounds could bind to the Q_i_ site more strongly than others. The significance of this focused study is that it shows that the 3-aryl quinolone derivatives generally have lower off-target mammalian effects (ELQ300, WDH-1-U4) compared with 2-aryl quinolones substituted with similar aryl sidechains. The outlier to this observation is SCR0911, which exemplifies that a curved 2-substituted sidechain can improve the selectivity and provides a template for further targeted studies for lead optimization.

Sequence differences in the C-terminal region of the E-ef loop of cytochrome *b* (notably the loss of histidine and lysine residues in the parasite), a region of the protein adjacent to the catalytically essential ef helix element of Q_o_, may also help drive drug selectivity [4,73]. It is predicted that the larger binding pocket of the Q_o_ site can accommodate a greater frequency of mutations than the Q_i_ site without a deleterious effect on enzyme turnover. This is in part supported by the observation that the propensity for the parasite to develop (point-mutation-based) resistance to Q_i_ inhibitors, e.g., ELQ300, is far lower compared to Q_o_ inhibitors, e.g., atovaquone (the reported resistance frequencies were 1 in  10^5^ parasites for atovaquone [74] and less than 1 in 10^8^ parasites for ELQ300) [38]. This feature makes the Q_i_ site potentially an advantageous target over Q_o_ site for exploring selective inhibitor binding to the *P. falciparum* enzyme. The targeting of the Q_i_ site is additionally attractive for developing antimalarial against existing atovaquone-resistant parasites.

Currently, the structure of cytochrome *bc_1_* complex from malarial parasites is not available. Molecular docking of antimalarial artemisinins and 4(1H)-pyridones to their target models, including cytochrome *bc_1_*, can describe structure and activity relationship and suggest rational design for new antimalarial lead compounds [75,76,77]. To further explore our data, a homology model of *P. falciparum* cytochrome *bc_1_* (*Pf bc_1_*) was generated by SwissModel online platform [58,59,60]. The crystal structure of bovine cytochrome *b*, which has the highest identity in the primary sequence (42%) among the structures from other organisms, was used as a starting model. However, the docking model with flexible ligand, such as ELQ300, shown in the current study might be less accurate [63]. It is possible that ELQ300 forms a hydrogen bond with His 192 when the headgroup rotates around the oxygen linker atom.

This observation suggests that 2-bisaryl 4(1H)quinolones (CK-2-67 and RKA066) might also target the Q_o_ site in *Pf bc_1_*, while ELQ300, SCR0911, and WDH-1U-4 are much more selective for the Q_i_ site. In support of this hypothesis, a recent study describing the in vitro selection of CK-2-68, a CK-2-67 derivative and resistant parasites, reported mutations in the Q_o_ site [78]. We and other workers have suggested that, for certain substitution patterns, quinolones can bind to both Q_o_ and Q_i_ sites opening the possibility of a certain degree of cross resistance with atovaquone [44,70].

## 5. Conclusions

Structural studies of bovine cytochrome bc1 complexed with promising antimalarial 4(1H)-quinolones demonstrate that some 2-aryl and 3-aryl 4(1H)-quinolones are specific binders of the Qi site. The 4(1H)-quinolone core of each of the compounds studied (CK-2-67, RKA066, and WDH-1U-4) locates in the hydrophilic region at the apex of Qi pocket, while aryl sidechains extend away through the hydrophobic channel. Surprisingly, substitution of the 2- or 3-position of the 4(1H)-quinolone core leads to similar interactions with the Qi site with inversion of the quinolone head group direction within the hydrophilic apex of the Qi site. Through the detailed analysis of seven molecules covering lead compounds and drug candidates from the quinolone and pyridone families, it is proposed that the number of hydrogen bonds with Qi site residues is the most important factor contributing to strong inhibition of the bovine enzyme, as exemplified by RKA066 and GSK932121. In silico docking revealed that the flexibility of the tail group is an important factor in binding to the *P. falciparum bc1* complex. Rigidity of the aryl sidechain, particularly the curved phenyl-pyridine sidechain in SCR0911, could further enhance inhibition *Pfbc_1_* via pi-pi stacking interactions of Phe30 and Phe37 and thus represents an excellent scaffold for further optimization.

The structural differences of the ubiquinone-reduction (Qi) site between the human and *P. falciparum* provides new opportunities for structure-based lead optimization and drug design. Binding analyses of 4(1H)-quinolones in *P. falciparum* and human Qi sites generated from molecular docking and bovine crystal structures suggest several interactions that could be used for development of the next generation 4(1H)-quinolone lead compounds with improved selectivity against *P. falciparum* with reduced toxicity profiles.

## Figures and Tables

**Table 1:** Inhibition profiles of selected antimalarial compounds against whole cells of *Plasmodium falciparum* (*Pf*) (3D7 and TM90C2B strains) and individual mitochondrial enzymes.

Compound	IC_50_ (nM) (3D7)	IC_50_ (nM) (TM90C2B)	IC_50_ (nM) (*Pf*NDH_2_)	IC_50_ (nM) (*Pfbc_1_*) *	Bovine Cytochrome *bc_1_* Inhibition (%)	
					at 0.1 µM	at 1 µM
ELQ300	2.2	1.7	NA	0.56	22 ± 5	51 ± 7
GSK932121	6	6 ^†^	>10,000 *	7	65 ± 4	82 ± 5
GW844520	5	2 ^†^	>10,000 *	2	59 ± 6	79 ± 3
SCR0911	12 ± 3	7.2	>1000 *	80% inhibition at 100 nM *	9 ± 2	72 ± 5
CK-2-67	117 ± 27	122 ± 26	16	38	90 ± 3	95 ± 1
RKA066	22 ± 0.4	217 ± 18	55	28	81 ± 4	93 ± 2
WDH-1U-4	36 ± 6	5 ± 2	492	<10	7 ± 1	61 ± 3

3D7—drug sensitive *P. falciparum* parasite; TM90C2B—atovaquone resistant parasite; NA—not available from literature; * Single point concentration only, full IC_50_ not available. ^†^ FCR3A strain used as a atovaquone-resistant parasite. Sources of IC_50_ values against parasite cells: ELQ300 [38]; GSK932121 and GW844520 [34]; SCR0911 [32]; CK-2-67, RKA066, and WDH-1U-4 [10,46]. Sources of IC_50_ values against individual mitochondrial enzymes (NDH_2_ and cytochrome *bc_1_*): ELQ300 [38]; GSK932121 and GW844520 [34]; SCR0911 [32]; CK-2-67, RKA066, and WDH-1U-4 [10,46]. Bovine *bc_1_* inhibitions of all compounds and *Pf bc_1_* inhibition of SCR0911 were measured in this work.

**Table 2:** Data collection and refinement statistics.

	*bc_1_*-CK-2-67	*bc_1_*-RKA066	*bc_1_*-WDH-1U-4
**Data collection**			
Space group	P6_5_22	P6_5_22	P6_5_22
Cell dimensions			
*a*, *b*, *c* (Å)	209.59, 209.59, 342.42	210.74, 210.74, 343.94	209.56, 209.56, 343.36
α, β, γ(°)	90, 90, 120	90, 90, 120	90, 90, 120
Resolution (Å)	49.81–3.20 (3.27–3.20)	89.85–3.50 (3.60–3.50)	90.74–3.50 (3.60–3.50)
*R*_merge_	0.21 (1.63)	0.27 (1.42)	0.20 (0.87)
*R_pim_*	0.09 (0.69)	0.09 (0.49)	0.09 (0.40)
CC_1/2_	0.997(0.357)	0.987(0.450)	0.968(0.543)
*I*/σ	8.5 (1.7)	5.0 (1.3)	7.0 (1.8)
Completeness (%)	95.7 (97.2)	100 (100)	97.4 (98.7)
Redundancy	9.4 (8.8)	9.0 (8.8)	5.4 (5.5)
**Refinement**			
Resolution (Å)	49.86–3.20	89.85–3.50	90.74–3.50
No. reflections	66,560	54,431	52,188
*R*_work_/*R*_free_(%)	21.4/25.02	22.09/23.82	21.71/24.57
No. atoms			
Protein	16,142	15,688	15,617
Inhibitor	30	31	30
Other ligands	570	565	558
Water	41	8	36
*B*-factors (Å^2^)			
Protein	90.68	140.24	133.88
Inhibitor	110.02	83.02	142.60
Other ligands	110.02	151.01	149.60
Water	54.87	45.87	40.79
R.m.s. deviations			
Bond lengths (Å)	0.007	0.009	0.008
Bond angles (°)	1.681	1.505	1.437
Ramachandran Plot			
Preferred (%)	96.02	95.98	96.17
Allowed (%)	3.59	3.66	3.63
Outliers (%)	0.39	0.35	0.20
PDB code	7R3V	6ZFU	6ZFS

## Data Availability

The atomic coordinates and structure factors (PDB codes 7R3V for *bc_1_*-CK-2-67; 6QTH for *bc_1_*-RKA066; and 6QTE for *bc_1_*-WDH-1U-4) have been deposited in the Protein Data Bank (http://www.rcsb.org). Authors will release the atomic coordinates and experimental data upon article publication.

## Supplementary Materials

The following supporting information can be downloaded at: https://www.mdpi.com/article/10.3390/biology11081109/s1, Experimental details of molecular docking to Pf bc_1_, docking scores, and additional figures illustrating reliability of docking method (PDF). Molecular format strings and some biological data (CSV). Homology model of Plasmodium falciparum cytochrome b subunit (PDB).

## Abbreviations

Å: angstrom; *bc_1_*, cytochrome *bc_1_*; DEAE, diethylaminoethyl cellulose; DMSO, dimethyl sulfoxide; PEG, polyethylene glycol; F_o_, observed structure factor; F_c_, calculated structure factor; KPi, potassium phosphate buffer; *Pf*, *Plasmodium falciparum*; Q_o_, ubiquinol-oxidation site; Q_i_, ubiquinone-reduction site.
