# Supplementary material for: Targeting the Ubiquinol-Reduction (Qi) Site of the Mitochondrial Cytochrome bc1 Complex for the Development of Next Generation Quinolone Antimalarials
Source: Biology (Basel). 2022 Jul 25;11(8):1109. doi: 10.3390/biology11081109 (PMC9330653; doi:10.3390/biology11081109)
Supplement: Supplementary file 1 [file biology-11-01109-s001.zip › Biology_1733521_SI-further question_sv.pdf]

biology

MDPI

Supplementary Material

Targeting the Ubiquinol-Reduction (Q_i) Site of the Mitochondrial Cytochrome *bc*$_1$ Complex for the Development of Next Generation Quinolone Antimalarials

Kangsa Amporndanai [1,2,†], Nattapon Pinthong [1,3,†], Paul M. O'Neill [4,*], W. David Hong [4], Richard K. Amewu [4,5], Chandrakala Pidathala [4,6], Neil G. Berry [4], Suet C. Leung [4], Stephen A. Ward [7], Giancarlo A. Biagini [7], S. Samar Hasnain [1] and Svetlana V. Antonyuk [1,*]

1 Molecular Biophysics Group, Institute of Systems, Molecular and Integrative Biology, Faculty of Health and Life Sciences, University of Liverpool, Liverpool L69 7ZB, UK; kangsa.amporndanai@Vanderbilt.Edu (K.A.); n.pinthong@liverpool.ac.uk (N.P.); s.s.hasnain@liverpool.ac.uk (S.S.H.)
2 Department of Biochemistry, Vanderbilt University School of Medicine, Nashville, TN 37232-0146, USA
3 Department of Protozoology, Faculty of Tropical Medicine, Mahidol University, Bangkok 10400, Thailand
4 Department of Chemistry, University of Liverpool, Liverpool L69 7ZD, UK; davidhwq@liverpool.ac.uk (W.D.H.); amewu@ug.edu.gh (R.K.A.); drchandrakalapidathala@cimedlife.com (C.P.); ngberry@liverpool.ac.uk (N.G.B.); scleung@liverpool.ac.uk (S.C.L.)
5 Department of Chemistry, School of Physical and Mathematical Sciences, University of Ghana, P.O. Box LG 586, Accra, Ghana
6 Composite Interceptive Med-Science Laboratories Pvt. Ltd., Bengaluru 60099, Karnataka, India
7 Centre for Drugs and Diagnostics, Tropical Disease Biology, Liverpool School of Tropical Medicine, Liverpool L3 5QA, UK; steve.ward@lstmed.ac.uk (S.A.W.); giancarlo.biagini@lstmed.ac.uk (G.A.B.)
* Correspondence: pmoneill@liverpool.ac.uk (P.M.O.); s.antonyuk@liverpool.ac.uk (S.V.A.); Tel.: +44-(0)-1517955145 (S.V.A.); +44-(0)-1517943552 (P.M.O.)
† These authors contribute equally to this work.

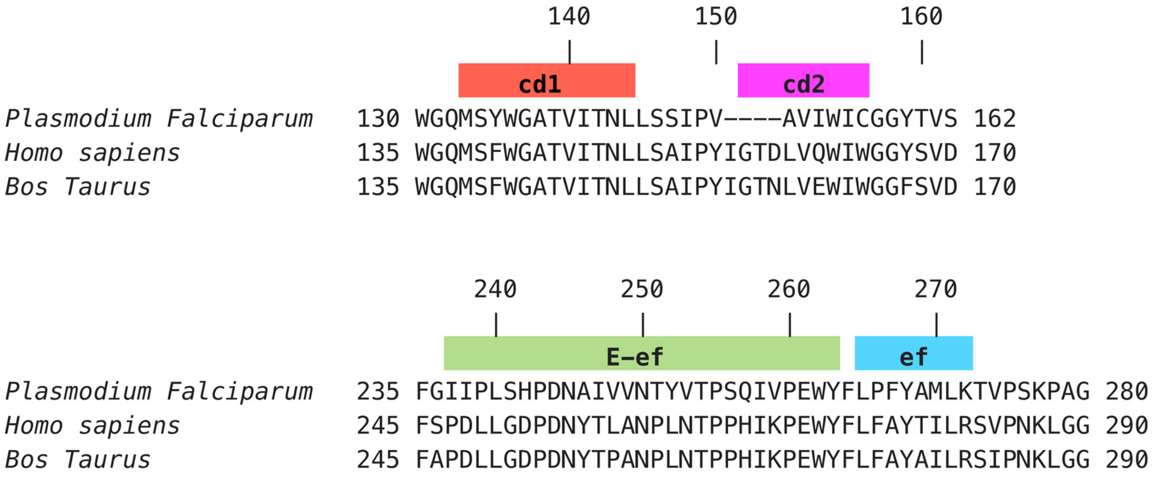

Figure S1. Sequence alignment of cytochrome *b* from *Plasmodium falciparum*, human and bovine at the regions of E-ef loop, cd1, cd2 and ef helices.

Molecular Docking

Chemical structures of inhibitors were drawn by using MarvinSketch ChemAxon version 15.9.28.0 software and saved in mol2 format. The homology model and inhibitor structures were submitted to SwissDock online platform to perform molecular docking using EADock dihedral space sampling algorithm [1]. A binding pocket of the Q_i site was defined within 25x25x25Å box centred at the position of N_e of His192 for *Pf* homology model and at the position of N

of His201 in the bovine structure. The parameter selected for docking on the SwissDock server was "accurate" allowing 5Å flexibility of side chain of any amino acids in the active site. The binding energies and scores were calculated using CHARMM (Chemistry at HARvard Macromolecular Mechanics) forcefield [2]. The predicted binding modes of the ligand are ranked according to their *FullFitness* scores. A more favourable binding mode is indicated by negativity associated with *FullFitness* score. All docking solutions were visualized by UCSF-Chimaera software for investigation and evaluation. Multiple conformations (255-260 results) for each inhibitor in the Q_i site were obtained from SwissDock and evaluated by two criteria to select the most possible solution. First, the placement of inhibitor in the docked model was compared to corresponding inhibitor-bound bovine cytochrome *bc₁* crystal structure if available. The conformations of the ligand that pose in different direction from the ligand in the crystal structure or locate outside the Q_i pocket were eliminated. Second, a solution with the highest rank of *Fullfitness* score was selected for the final docking solution. *Fullfitness* scores and calculated binding Gibbs free energy of each inhibitor are shown in Table S1. The negative Gibbs free energies indicate that the protein-ligand complexes are plausible.

To show reliability of docking method, GSK932121, SCR0911, CK-2-67, RKA066 and WDH-1U-4 were docked to the bovine cytochrome *bc_1* structures (Table S2). All bovine *bc_1* docking results were compared with the conformations found in bovine cytochrome *bc_1* structures. Root mean square derivation (r.m.s.d.) values were determined using UCSF-Chimaera software[3] indicating low difference between experimental and predicted binding poses, with r.m.s.d. values less than 2 Å (Figure S3). The ability to reproduce co-crystallographic bovine enzyme experimental data using the *in-silico* experiments provides confidence for the predicted conformations of lead compounds for inhibitor binding poses in *Pf* cytochrome *bc_1*.

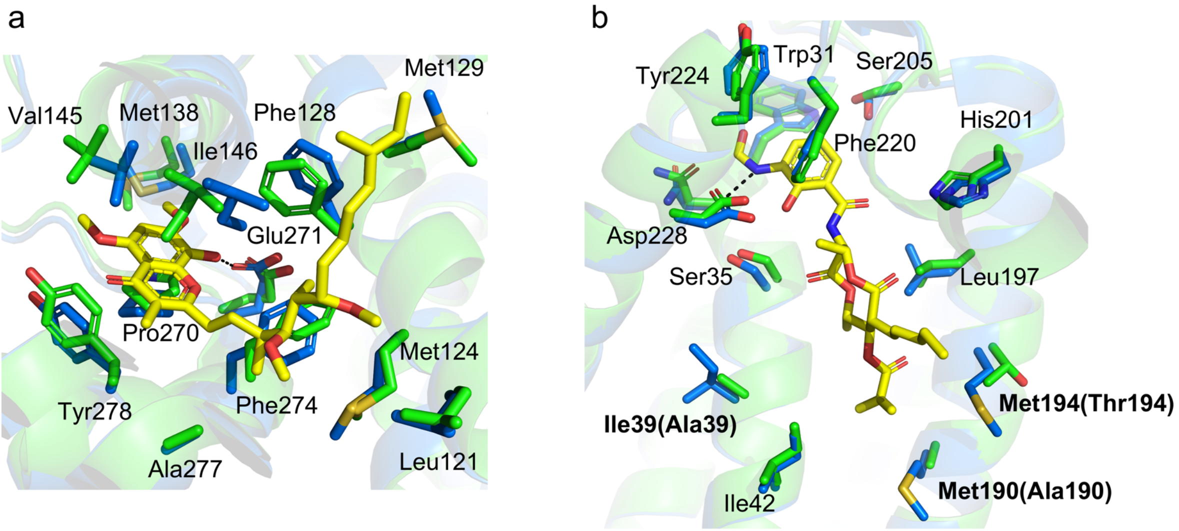

Figure S2. Superimposed crystal structures of bovine (PDB: 1PPJ, blue) and human (PDB:5XTE, green) cytochrome *bc_1*. (a) Q_o site occupied by stigmatellin A. (b) Q_i site occupied by antimycin A. Bovine residues that differ from host are labelled in bold with the human residues in the brackets. Inhibitors are shown as yellow sticks. Hydrogen bonds are shown as black dashed lines.

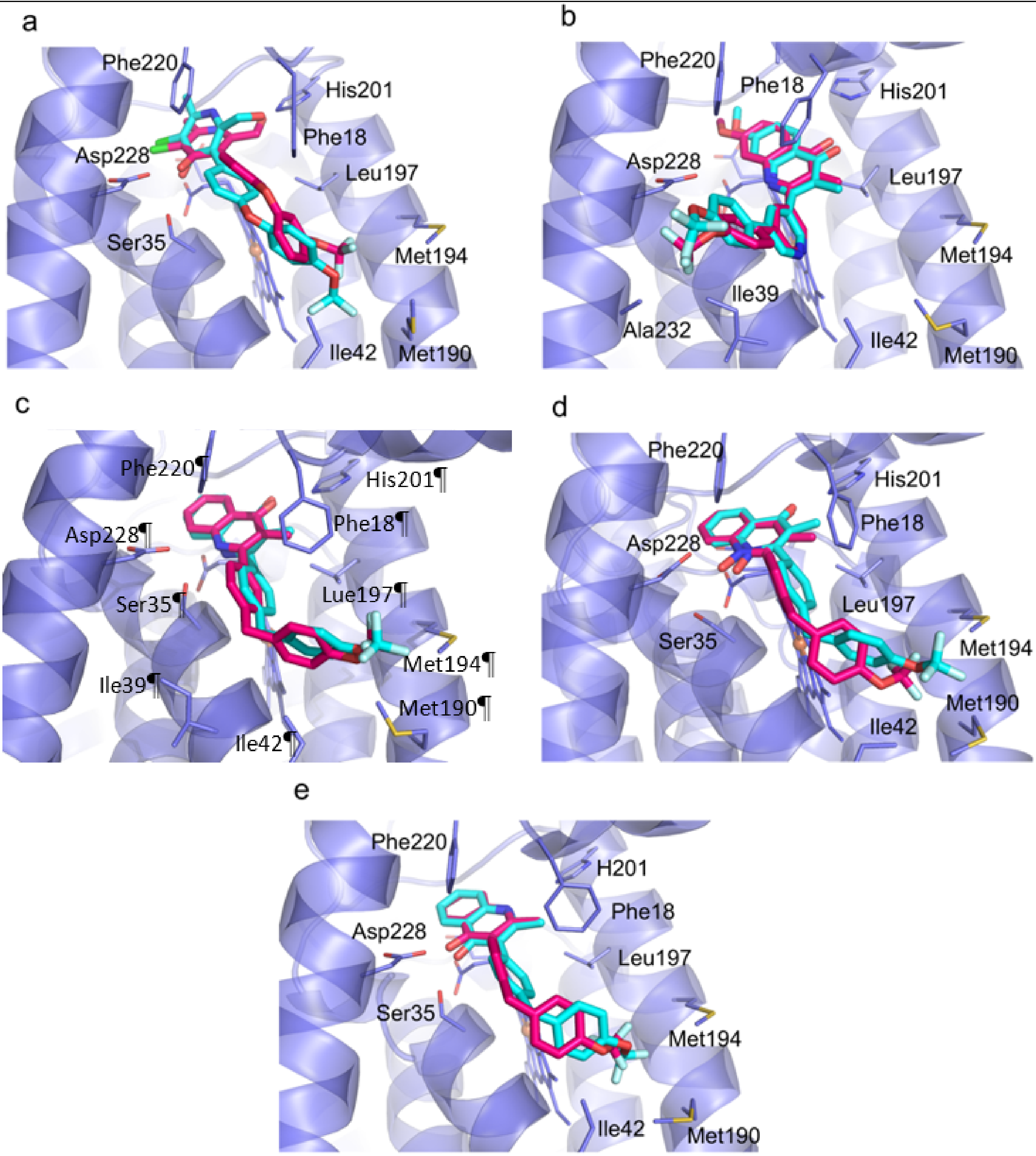

Figure S3. Comparison of the inhibitor docking poses to bovine Q_i site and their conformations obtained by X-ray crystallography. (a) GSK932121 (r.m.s.d. 1.73Å) (b) SCR0911 (r.m.s.d. 0.79Å) (c) CK-2-67 (r.m.s.d. 1.39Å) (d) RKA066 (r.m.s.d. 1.60Å) (e) WDH-1U-4 (r.m.s.d. 0.71Å). Inhibitor molecules are shown as sticks and colored by atom types (carbon of docking molecule in cyan; carbon of crystal structure in pink; oxygen in red; chlorine in green, fluorine in light blue). Cytochrome *b* subunit and Q_i site residues are shown as blue cartoon and sticks, respectively.

Table S1. Docking *Fullfitness* scores and calculated Gibbs free energies of molecular docking to *Pf* homology model.

Compound	*Fullfitness* score (kcal/mol)	Gibbs free energy (kcal/mol)
ELQ300	-828.42	-8.73
GSK932121	-828.01	-8.11
SCR0911	-837.06	-7.68
CK-2-67	-844.30	-7.87
RKA066	-839.40	-7.89
WDH-1U-4	-837.35	-8.22

Table S2. Docking *Fullfitness* scores and calculated Gibbs free energies of molecular docking to bovine crystal structures.

Compound	*Fullfitness* score (kcal/mol)	Gibbs free energy (kcal/mol)
GSK932121	-860.81	-7.61
SCR0911	-821.12	-8.21
CK-2-67	-834.78	-7.58
RKA066	-829.27	-8.34
WDH-1U-4	-822.14	-8.05

References

1. Grosdidier, A.; Zoete, V.; Michielin, O. SwissDock, a protein-small molecule docking web service based on EADock DSS. *Nucleic Acids Res.* **2011**, *39*, W270–W277. https://doi.org/10.1093/nar/gkr366.
2. Grosdidier, A.; Zoete, V.; Michielin, O. Fast docking using the CHARMM force field with EADock DSS. *J. Comput. Chem.* **2011**, *32*, 2149–2159. https://doi.org/10.1002/jcc.21797.
